# A new species of *Polypedilum* (*Cerobregma*) (Diptera, Chironomidae) from Oriental China

**DOI:** 10.3897/zookeys.1011.59554

**Published:** 2021-01-22

**Authors:** Wen-Bin Liu, Yuan Yao, Chun-Cai Yan, Xin-Hua Wang, Xiao-Long Lin

**Affiliations:** 1 Tianjin Key Laboratory of Conservation and Utilization of Animal Diversity, Tianjin Normal University, Tianjin, 300387, China Tianjin Normal University Tianjin China; 2 College of Life Sciences, Nankai University, Tianjin, 300071, China Nankai University Tianjin China

**Keywords:** COI, Chironomini, integrative taxonomy, new species

## Abstract

Polypedilum (Cerobregma) huapingensis Liu & Lin, **sp. nov.** is described and illustrated based on an adult male from Huaping National Nature Reserve, Guangxi, China. A DNA barcode analysis, including the known partial COI sequences of species in the *Cerobregma* subgenus, was conducted. An updated key to adult males of the subgenus Cerobregma is provided.

## Introduction

The genus *Polypedilum* Kieffer is one of the largest chironomid genera, containing eight subgenera and more than 520 described species (Sæther et al. 2010; Cranston et al. 2016; Yamamoto et al. 2016; P. Ashe, pers. comm.). The larvae mostly occur in sediments, but some species are associated with mines of aquatic plants or co-inhabit pupal retreats of caddisflies (Cranston et al. 1989). Adult males of the subgenus Cerobregma Sæther & Sundal, 1999 are characterized by having extremely long and strong, split setae along the inner margin of the gonostylus and gonocoxite, with an apicolateral bulb-like extension with deep lateral incision between the bulb and the gonostylus. The subgenus Cerobregma includes 15 valid species recorded in the Afrotropical, Holarctic and Oriental regions (Tokunaga 1940; Sæther and Sundal 1999; Kobayashi et al. 2003; Zhang and Wang 2005; Zhang et al. 2006; Moubayed-Breil 2007; Tang and Niitsuma 2017; Lin et al. 2019).

The DNA barcode corresponding to the 658-bp fragment of the mitochondrial gene cytochrome c oxidase I (COI) has been identified as the core of a global bio-identification system at the species level (Hebert et al. 2003a, b) and has proved to be useful in non-biting midge species delimitation (Anderson et al. 2013; Silva et al. 2014; Lin et al. 2015; Montagna et al. 2016; Giłka et al. 2018; Lin et al. 2018; Qi et al. 2017). COI barcodes have provided important evidence to confirm new species descriptions within Polypedilum species (Song et al. 2016, 2018; Lin et al. 2019).

The Nanling-Mountain region, located in the middle subtropical zone of China, rich in biological resources and with a warm and moist climate, is a typical natural ecosystem and one of the most biologically diverse areas in the world. Recently, during investigations of insect diversity in the Nanling Mountains, we discovered an unknown species of the subgenus Cerobregma from Huaping National Nature Reserve. In the present study, Polypedilum (Cerobregma) huapingensis Liu & Lin sp. nov. is described and delimited by its morphology and DNA barcode. An updated key to adult males of the subgenus is provided.

## Materials and methods

The single specimen of the new species, collected by a Malaise trap, was preserved in 85% ethanol and stored in the dark at 4 °C before morphological and molecular analyses. Genomic DNA was extracted from the thorax and head using a Qiagen DNA Blood and Tissue Kit at Nankai University, Tianjin, China, following the standard protocol except for the final elution volume of 100 µl. After DNA extraction, the exoskeleton of each specimen was mounted in Euparal on a microscope slide together with the corresponding wings, legs, antennae and abdomen, following the procedures outlined by Sæther (1969). Morphological terminology follows Sæther (1980).

The color pattern of new species is described based on the specimen preserved in ethanol. Digital photographs of slide-mounted specimens were taken with a 300-dpi resolution using a Nikon Digital Sight DS-Fil camera mounted on Nikon Eclipse 80i compound microscope using the software NIS-Elements F v.4.60.00. at the College of Life Sciences, Nankai University, Tianjin, China.

The universal primers LCO1490 and HCO2198 (Folmer et al. 1994) were used to amplify the standard 658-bp mitochondrial COI barcode region. Polymerase chain reaction (PCR) amplifications followed Song et al. (2018) and were conducted in a 25 μl volume including 12.5 μl 2× Es Taq MasterMix (CoWin Biotech Co., Beijing, China), 0.625 μl of each primer, 2 μl of template DNA and 9.25 μl of deionized H_2_O. PCR products were electrophoresed in 1.0% agarose gel, and purified and sequenced in both directions at Beijing Genomics Institute Co. Ltd., Beijing, China.

Raw sequences were assembled and edited in Geneious Prime 2020 (Biomatters Ltd., Auckland, New Zealand). Alignment of the sequences was carried out using the MUSCLE algorithm (Edgar 2004) on amino acids in MEGA 6 (Tamura et al. 2013). The pairwise distances using the Kimura 2-Parameter (K2P) substitution model of six species within the subgenus Cerobregma were calculated in MEGA. The neighbor-joining tree was constructed using the K2P substitution model, 1000 bootstrap replicates and the “pairwise deletion” option for missing data in MEGA. Novel sequences, trace-files, and metadata of the new species were uploaded to the Barcode of Life Data Systems (BOLD) (Ratnasingham and Hebert 2013). The GenBank accession number for the new species is MW472357.

The holotype of the new species is deposited at the College of Life Sciences, Nankai University, Tianjin, China (**NKU**).

## Results

### DNA barcode analysis

**Figure 1.**
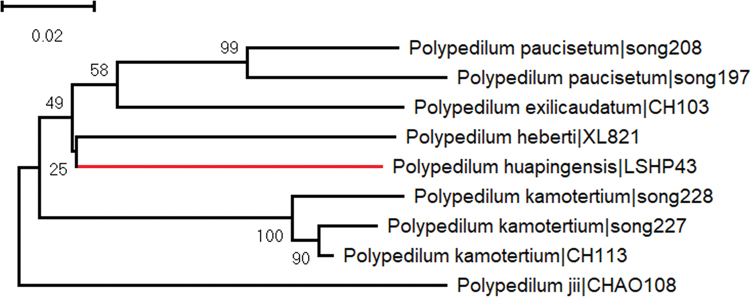
Neighbor-joining tree for six species of the subgenus Cerobregma based on K2P distance in DNA barcodes. Numbers on branches represent bootstrap support (>70%) based on 1000 replicates; scale equals K2P genetic distance.

The neighbor joining tree based on COI DNA barcodes of the six sequenced species within the subgenus Cerobregma revealed six distinct genetic clusters, suggesting one species new to science (Fig. 1). In some barcode studies in Chironomidae, the average threshold intraspecific divergence is 4–5% in *Tanytarsus* van der Wulp (Lin et al. 2015) and 5%–8% in *Polypedilum* Kieffer (Song et al. 2016, 2018). Polypedilum (Cerobregma) huapingensis sp. nov. can be differentiated from the other sequenced species by a more than 13% divergence in the COI barcode sequence (Tab. 1; Fig. 1).

**Table 1.** Kimura 2-parameter pairwise genetic distances based on COI barcodes of the Polypedilum (Cerobregma).

Species	Specimen reference number	Pairwise genetic distances								GenBank accessions
*P. exilicaudatum*	CH103									MG950021
*P. yamasinense*	song227	0.155								MG949754
*P. yamasinense*	CH113	0.147	0.015							MG949955
*P. jii*	CHAO108	0.185	0.168	0.156						MG950056
*P. yamasinense*	song228	0.155	0.041	0.034	0.179					MG950029
*P. paucisetum*	song208	0.117	0.161	0.147	0.171	0.157				MG950008
*P. paucisetum*	song197	0.138	0.163	0.153	0.162	0.170	0.075			MG949790
*P. heberti*	XL821	0.140	0.146	0.137	0.174	0.157	0.142	0.155		MK505566
*P. huapingensis*	LSHP43	0.130	0.145	0.136	0.173	0.153	0.140	0.159	0.135	MW472357

### Taxonomy

#### Polypedilum (Cerobregma) huapingensis

Taxon classificationAnimalia

Liu & Lin sp. nov.

7AF53580-6710-51FC-8F0D-C6E99E02E093

http://zoobank.org/79EB5D46-B309-4459-8D2C-903589EC65F3

Figures 2 , 3 , 4

##### Type material.

***Holotype***: male (NKU & BOLD sample ID: LSHP43), China, Guangxi Zhuang Autonomous Region, Guilin City, Lingui County, Huaping National Nature Reserve, 25.563°N, 109.942°E, 1271 m a.s.l., 10–20.VI.2020, Malaise trap, S.G. Zhao.

##### Etymology.

The specific name refers to the Huaping National Nature Reserve, where the holotype was collected.

##### Diagnostic characters.

According to the morphological characters of the adult male, the new Polypedilum species keys to the subgenus Cerobregma, and can be distinguished from other known species of the subgenus by the following combination of characters: tergites III–VI brown with dark brown spots at middle; wing pale brown with a large black spot on entire basal area of wing; superior volsella with basal microtrichia and two inner setae; anal point strong, contracted in middle, a large inflated globe apically with candle-like spine.

##### Adult male

**(n = 1).** Total length 4.29 mm. Wing length 2.71 mm. Total length/wing length 1.58. Wing length/length of profemur 1.57.

**Figure 2.**
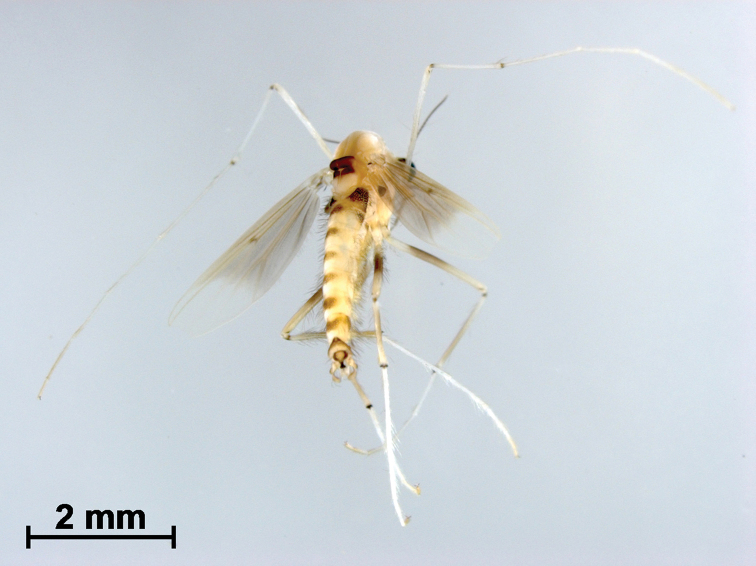
Polypedilum (Cerobregma) huapingensis Liu & Lin, sp. nov., holotype male.

***Coloration*** (Fig. 2). Head brown. Antenna yellow. Thorax ground color brown with dark brown stripes on scutum, laterally under parapsidal suture, postnotum and on preepisternum. Tergites III–VI brown with dark brown spots at middle; tergites I, II, VII, and VIII and hypopygium largely dark brown. Most of femora and tibiae dark brown, all tarsomeres yellow. Wing pale brown with a large black spot on entire basal area.

**Figure 3.**
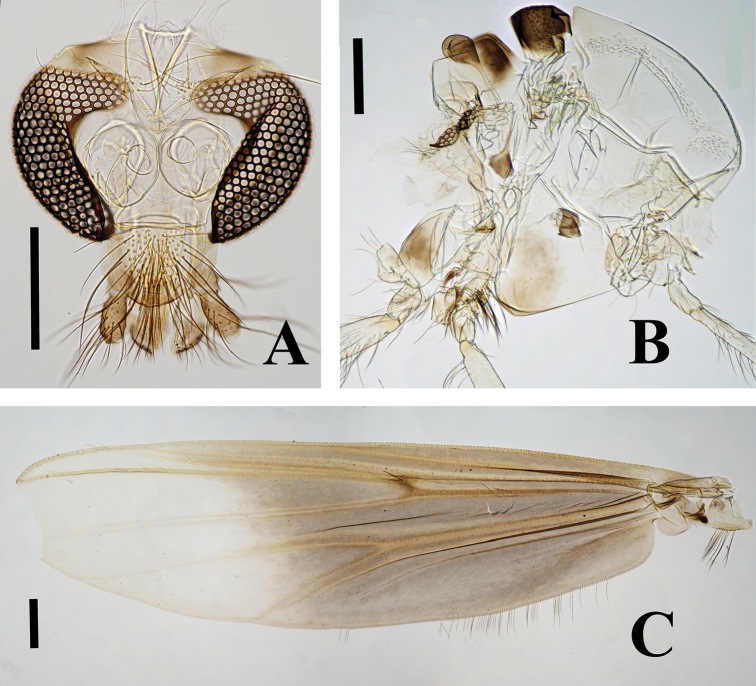
Polypedilum (Cerobregma) huapingensis Liu & Lin, sp. nov., holotype male **A** head **B** thorax **C** wing. Scale bars: 200 µm.

***Head*** (Fig. 3A). Antenna with 13 flagellomeres; ultimate flagellomere 425 µm long; AR 0.44. Temporal setae 27, including 7 inner verticals and 20 outer verticals. Clypeus with 79 setae. Tentorium 132 µm long; 45 µm wide. Stipes 112 µm long, 24 µm wide. Lengths of palpomeres 1–5 (in µm): 52.5, 97.5, 192, 170, 232. Palpomere ratio (5^th^/3^rd^) 1.21.

***Thorax*** (Fig. 3B). Acrostichals 31; humerals 38; dorsocentrals 104; prealars 16. Scutellum with 86 setae.

***Wing*** (Fig. 3C). VR 1.15. Brachiolum with 13 setae; R with 36 setae; R_1_ with 32 setae; R_4+5_ with 36 setae; M with 22 setae; remaining veins bare. R_2+3_ distinct. Squama with 16 setae. Anal lobe moderately developed.

***Legs*.** Spur of mid tibia 28 µm long, mid tibia including 55 µm long comb, un-spurs comb 35 µm long; spur of hind tibia 36 µm long including 78 µm long comb, un-spurs comb 40 µm long. Apical width of fore tibia 60 µm; of mid tibia 76 µm; of hind tibia 80 µm. Lengths (in µm) and proportions of legs as in Table 2.

**Table 2.** Lengths (in µm) and proportions of legs of Polypedilum (Cerobregma) huapingensis sp. nov., male holotype (n = 1).

	fe	ti	ta_1_	ta_2_	ta_3_	ta_4_	ta_5_	LR	BV	SV	BR
P_1_	1725	1108	2405	1025	725	625	275	2.17	2.11	1.98	3.14
P_2_	1702	1225	655	475	355	175	148	0.53	2.67	3.11	4.50
P_3_	1850	1208	1075	925	503	362	154	0.89	2.34	2.13	4.90

**Figure 4.**
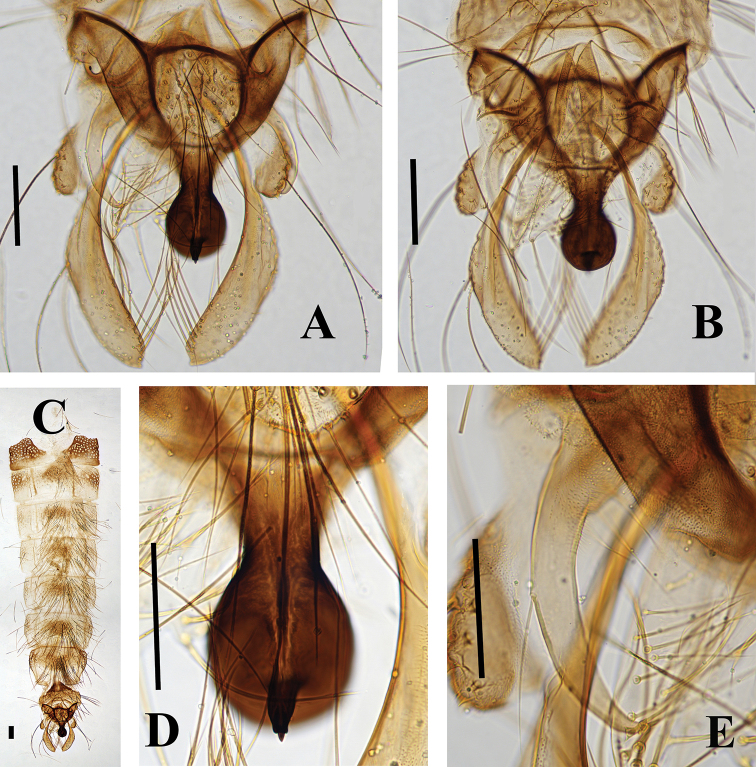
Polypedilum (Cerobregma) huapingensis Liu & Lin, sp. nov., holotype male **A** hypopygium, dorsal view **B** hypopygium, ventral view **C** abdomen **D** superior volsella **E** anal point. Scale bars: 100 µm.

***Hypopygium*** (Fig. 4). Anal tergite with 46 median setae. Laterosternite IX with seven setae. Anal point as in Fig. 4D, strong, contracted in middle, as a large inflated globe apically with a single candle-like spine, tapering, 143 µm long. Transverse sternapodeme 93 µm long; phallapodeme 168 µm long. Gonocoxite 320 µm long. Superior volsella 143 µm long, with basal microtrichia and two inner setae (Fig. 4E). Inferior volsella 164 µm long, finger-shaped, divided into two lobes apically, with 24 long setae. Gonostylus 310 µm long. HR 1.03; HV 1.38.

Female and immatures unknown.

##### Discussion.

The characters of the anal point and superior volsella of the new species place it within the subgenus Cerobregma. The morphology of the new species resembles that of *Polypedilum heberti* Lin &Wang, 2019, but it can be separated from it on the basis of the following: 1) tergites III–VI brown with dark brown spots at middle in the new species, versus tergites III–VI with dark brown bands at middle in *P. heberti*; 2) thorax of the new species (acrostichals 31; humerals 38; dorsocentrals 104) with much more setae than in *P. heberti* (acrostichals 8; humerals 5; dorsocentrals 20); 3) anal point strong and tapering in *P. heberti*, versus constricted in middle, with a large inflated globe apically with candle-like spine in the new species.

### Updated key to known adult males of Polypedilum (Cerobregma)

The following key replaces couplet 5 in Lin et al. (2019) and adds a couplet 5a to include the male of the newly described species.

**Table:** 

5	Wing with several spots; setae along inner margin of gonostylus strongly split	***P. ramiferum* Kieffer, 1921**
–	Wing with a large black spot on entire basal area; setae along inner margin of gonostylus not split	**5a**
5a	Acrostichals 8; humerals 5; dorsocentrals 20; anal point strong and tapering	***P. heberti* Lin & Wang, 2019**
–	Acrostichals 31; humerals 38; dorsocentrals 104; anal point strong, contracted in middle, as a large inflated globe apically with candle-like spine	***P. huapingensis* Liu & Lin, sp. nov.**

## Supplementary Material

XML Treatment for Polypedilum (Cerobregma) huapingensis
